# Supplementary material for: Creep Feeding and Weaning Influence the Postnatal Evolution of the Plasma Metabolome in Neonatal Piglets
Source: Metabolites. 2023 Jan 31;13(2):214. doi: 10.3390/metabo13020214 (PMC9960666; doi:10.3390/metabo13020214)
Supplement: Supplementary file 1 [file metabolites-13-00214-s001.zip › metabolites-2185227-supplementary - Copy.pdf]

metabolites

Article

Creep Feeding and Weaning Influence the Postnatal Evolution of the Plasma Metabolome in Neonatal Piglets

Barbara U. Metzler-Zebeli [1,2,*], Frederike Lerch [1,2], Fitra Yosi [1,2,4], Julia Vötterl [1,2], Simone Koger [2,5], Markus Aigensberger [2,3], Patrick M. Rennhofer [2,3], Franz Berthiller [2,3], Heidi E. Schwartz-Zimmermann [2,3],

1 Unit of Nutritional Physiology, Department of Biomedical Sciences, University of Veterinary Medicine Vienna, 1210 Vienna, Austria
2 Christian-Doppler Laboratory for Innovative Gut Health Concepts of Livestock, University of Veterinary Medicine Vienna, 1210 Vienna, Austria
3 Department of Agrobiotechnology (IFA-Tulln), Institute of Bioanalytics and Agro-Metabolomics, University of Natural Resources and Life Sciences, Vienna (BOKU), 3430 Tulln an der Donau, Austria
4 Department of Animal Science, Faculty of Agriculture, University of Sriwijaya, Palembang, South Sumatra, Indonesia
5 Department for Farm Animals and Veterinary Public Health, Institute of Animal Nutrition and Functional Plant Compounds, University of Veterinary Medicine Vienna, 1210 Vienna, Austria
* Correspondence:

Citation: Metzler-Zebeli, B.U.; Lerch, F.; Yosi, F.; Vötterl, J.C.; Koger, S.; Aigensberger, M.; Rennhofer, P.M.; Berthiller, F.; Schwartz-Zimmermann, H.E. Creep Feeding and Weaning Influence the Postnatal Evolution of the Plasma Metabolome in Neonatal Piglets. *Metabolites* **2023**, *13*, 214. https://doi.org/10.3390/metabo13020214

Academic Editor: Shozo Tomonaga

Received: 11 January 2023
Revised: 22 January 2023
Accepted: 29 January 2023
Published: 31 January 2023

Copyright: © 2023 by the authors. Licensee MDPI, Basel, Switzerland. This article is an open access article distributed under the terms and conditions of the Creative Commons Attribution (CC BY) license (https://creativecommons.org/licenses/by/4.0/).

Supplementary Materials

Table S1. Analysed nutrient composition of the piglets and sow diet.*

Chemical composition, % DM	Lactation Diet	Creep Feed	Prestarter Diet
Dry matter, %	89.0	94.8	91.9
Crude ash	5.5	5.6	5.5
Crude protein	17.9	19.8	20.5
Crude fibre	5.8	0.4	5.2
Neutral-detergent fibre	17.3	3.0	15.2
Acid-detergent fibre	6.7	0.6	5.8
Acid-detergent lignin	1.8	0.3	1.5
Crude fat	5.2	7.8	7.5
Nitrogen-free extract	65.7	66.5	61.6
Starch	47.3	29.4	31.9
Sugar	5.5	26.8	14.2
Metabolizeable energy, MJ/kg	14.7	16.9	15.3

* ZuchtsauenKorn S Vital, Garant-Tiernahrung GmbH, Pöchlarn, Austria. Ingredient composition: corn, wheat, barley, soybean meal, sunflower meal, wheat bran, apple pomace, soybean oil, calcium carbonate, monocalcium phosphate, sodium chloride, magnesium phosphate, fish oil, L-cellulose, molasses. Vitamin and mineral composition per kg feed: 10,000 IU of vitamin A, 1,800 IU of vitamin D, 100 mg of Fe as iron(II) sulfate, 15 mg of Cu as copper(II) sulfate, 90 mg of Zn as zinc sulfate, 40 mg of Mn as manganese(II) oxide, 1.5 mg of I as calcium iodate, 0.4 mg of Se as sodium selenite. Technological additives: 500 FTU phytase, 2 mg of butylated hydroxyanisole, 10 mg of butylated hydroxytoluene, 2 mg of propyl gallate.

Weanplus-4, Startix, Voorthuizen, The Netherlands. Ingredient composition: whey powder, starch, soy protein, plant protein, plant oil (coconut oil and palm oil). Vitamin and mineral composition per kg feed: 25,000 IU of vitamin A, 8,000 IU of vitamin D3, 200 mg of vitamin E, 140 mg of Cu as chelate of glycine hydrate, 108 mg of Fe as chelate of glycine hydrate, 3 mg of I as calcium iodate, 115 mg of Zn as chelate of glycine hydrate, 35 mg of Mn as glycine chelate, 0.3 mg of Se as sodium selenite. Technological additives: 5 g of citric acid, 1 g of calcium formiate. Mixing ratio: 200 g of powder mixed into 1 L of 45°C warm water.

Ferkelabsetzkorn OGT, Garant-Tiernahrung GmbH, Pöchlarn, Austria. Ingredient composition: Oat flakes, barley, wheat, whey powder, soy protein concentrate, wheat bran, sucrose, soybean meal, soy oil, corn gluten, L-cellulose, monocalcium phosphate, fish oil, sodium chloride, magnesium phosphate, calcium carbonate and molasses. Vitamin and mineral composition per kg feed: 16,000 IU of vitamin A, 2,000 IU of vitamin D_3, 200 mg vitamin E, 120 mg Fe as iron(II) sulfate, 140 mg Cu as copper(II) sulfate, 120 mg Zn as zinc sulfate, 60 mg Mn as manganese(II) oxide, 1.5 mg I as calcium iodate, 0.5 mg Se as sodium selenite. Technological additives: 1,000 IU of phytase, 1,500 EPU of xylanase, 11 mg of beta hydroxy acid, 21 mg of butylated hydroxytoluene, 11 mg of propyl gallate.

Table S2. Reversed phase high-performance liquid chromatographic methods.*

	**sSRM Positive Ionization Mode**		**SRM Negative Ionization Mode**		**SRM Positive Ionization Mode (Lipids)**	
Mobile phase A	Water/formic acid (99.8/0.2, v/v)				Water/formic acid (99.9/0.1, v/v) +10 mM ammonium formate	
Mobile phase B	ACN/formic acid (99.8/0.2, v/v)				Water/i-propanol/ACN/formic acid (27.9/417.0/555.0/0.1, v/v/v/v) +10 mM ammonium formate	
Gradient	Time (min)	% B	Time (min)	% B	Time (min)	% B
	0.0	5	0.0	10	0.0	25
	0.3	5	0.3	10	0.8	25
	2.7	15	2.0	25	3.5	70
	4.0	40	4.9	37	7.0	100
	5.0	100	6.0	50	10.5	100
	5.2	100	6.5	100	10.6	25
	6.0	100	6.6	100	12.0	25
	6.1	5	7.8	100		
	7.5	5	7.9	10		
			9.0	10		
Injection volume	2 µL		5 µL		2 µL	

*ACN, acetonitrile; sSRM, scheduled selected reaction monitoring; SRM, selected reaction monitoring.

Table S3. Selected reaction monitoring (SRM) transitions for liquid chromatography - tandem mass spectrometric measurements in positive ionization mode for non-lipid compounds and their internal standards.*

**Positive Ionization Mode, Non-Lipid Compounds**

Amino Acid-Related Compounds				
**Analyte**	**Precursor Ion (*m*/*z*)**	**Declustering Potential (V)**	**Product Ions (quant/qual, *m*/*z*)**	**CE (quant/qual, eV)**
N-methylalanine	104.0	50	58.0/42.0	20/60
Betaine	118.1	70	58.0/59.0	35/25
Creatine	132.1	50	90.0/44.0	20/45
Stachydrine	144.0	81	84.0/144.0	31/5
3-Indoleacetic acid	176.1	60	130.0/103.0	25/45
Hippuric acid	180.1	50	105.0/77.0	13/45
Nicotinuric acid	181.1	60	135.1/79.0	25/60
3-Indolepropionic acid	190.1	60	130.0/172.0	25/20
Phenylacetylglycine	194.1	50	91.0/76.0	35/13
Beta-alanine	225.0	70	90.0/136.0	15/30
Sarcosine	225.0	70	90.0/136.0	15/30
Gamma-Aminobutyric acid	239.0	70	104.0/87.0	15/25
Alpha-aminobutyric acid	239.0	70	104.0/58.0	15/30
Beta-minobutyric acid	239.0	70	104.0/44.0	15/30
Tryptophan betaine	247.0	70	188.1/118.0	22/45
5-Aminovaleric acid	253.0	70	100.0/94.0	23/22
Homoserine	255.0	70	120.0/74.0	15/27
Trans-4-hydroxyproline	267.1	70	132.0/86.0	20/35
cis-4-Hydroxyproline	267.1	70	132.0/86.0	18/30
Alpha-aminoadipic acid	297.1	70	162.0/98.0	15/35
Methionine sulfoxide	301.2	70	237.0/88.0	20/40
1-Methylhistidine	305.1	70	124.0/83.1	30/55
3-Methylhistidine	305.1	70	168.0/212.0	30/25
Acetylornithine	310.2	70	217.0/175.0	15/25
Citrulline	311.2	70	70.1/159.0	55/25
Homoarginine	324.1	70	189.0/84.1	25/45
Dihydroxyphenylalanine	333.1	70	198.0/152.0	20/30
N,N-Dimethylarginine	338.2	70	203.0/46.1	25/62
N,N'-Dimethylarginine	338.2	70	203.0/172.0	25/30
Carnosine	362.2	70	207.0/269.0	25/20
Nitrotyrosine	362.2	70	181.0/227.0	30/20
Anserine	376.1	70	241.0/283.0	25/28
Ornithine	403.2	70	310.0/175.0	15/25
Homocysteine	406.0	70	237.0/88.1	20/55
**Amino Acids**				
**Analyte**	**Precursor Ion (*m*/*z*)**	**Declustering Potential (V)**	**Product Ions (quant/qual, *m*/*z*)**	**CE (quant/qual, eV)**
Glycine	211.2	70	76.0/136.0	15/25

Alanine	225.0	70	90.0/136.0	15/30
Serine	241.2	70	106.0/136.0	15/30
Proline	251.2	70	116.0/70.0	18/35
Valine	253.0	70	118.0/72.0	15/25
Threonine	255.2	70	120.0/74.0	15/30
Leucine	267.3	70	43.0/132.0	60/15
Isoleucine	267.3	70	69.0/86.0	40/20
Asparagine	268.2	70	251.0/87.0	15/25
Aspartic acid	269.2	70	136.0/88.0	30/25
Glutamine	282.2	70	130.0/84.1	25/38
Glutamic acid	283.2	70	148.0/84.0	15/35
Methionine	285.1	70	150.0/133.0	15/25
Histidine	291.1	70	198.0/110.0	15/30
Phenylalanine	301.2	70	120.0/166.0	25/15
Arginine	310.0	70	175.0/217.0	25/15
Tyrosine	317.2	70	182.0/136.0	15/30
Tryptophan	340.2	70	188.0/146.0	25/35
Cysteine	392.0	70	223.0/177.1	20/48
Lysine	417.2	70	324.0/189.0	20/25
**Amines**				
**Analyte**	**Precursor ion (*m*/*z*)**	**Declustering Potential (V)**	**Product Ions (quant/qual, *m*/*z*)**	**CE (quant/qual, eV)**
Trimethylamine	60.1	60	60.1/60.1	10.Okt
Trimethylamine-N-oxide	76.1	80	59.1/58.0	18/25
Choline	104.0	60	60.0/58.0	25/40
Creatinine	114.1	60	44.1/86.0	20/15
Benzamide	122.1	70	77.0/105.0	35/20
Trigonelline	138.0	95	92.0/94.0	30/35
Carnitine	162.1	60	103.0/85.0	25/30
Methylamine	167.0	70	94.0/77.0	20/40
Dimethylamine	181.0	70	88.0/136.0	20/25
Phosphocholine	184.1	70	156.0/120.0	15/20
Ethanolamine	197.1	70	136.0/62.0	25/18
Pyrrolidine	207.0	70	114.0/72.1	20/40
2-Hydroxypyridine	231.1	70	94.0/138.0	20/28
Histamine	247.0	70	154.0/95.0	18/35
2-Phenylethylamine	257.2	70	105.0/94.0	30/20
Taurine	261.0	70	126.0/136.0	20/30
Dopamine	289.2	70	137.0/91.0	25/52
Serotonin	312.3	70	160.0/115.1	30/65
Kynurenine	344.2	70	192.0/146.0	20/30

Putrescine	359.1	70	266.0/114.0	15/35
Cadaverin	373.1	70	280.0/128.0	20/35
Spermidine	551.2	70	193.1/416.2	40/20
Spermine	743.3	70	473.0/193.1	25/60
Thyroxine	912.7	70	777.5/731.6	30/50
		**Eicosanoids and Sterols**		
**Analyte**	**Precursor Ion (*m*/*z*)**	**Declustering Potential (V)**	**Product Ions (quant/qual, *m*/*z*)**	**CE (quant/qual, eV)**
Prostaglandin F2α	337.2	50	319.2/301.2	15/20
Cortexolone	347.2	75	109.1/311.2	50/25
Thromboxane B2	353.2	60	335.2/317.2	15/15
Cortisone	361.1	80	163.1/121.1	34/40
Cortisol	363.2	80	327.2/121.1	25/35
5-Dihydrocortisone	363.2	100	327.2/345.2	20/20
Prostaglandin E2	370.2	60	317.2/299.2	15/20
Prostaglandin D2	370.2	60	317.2/335.2	15/15
Prostaglandin E2 ethanolamide	378.3	80	360.3/342.3	15/20
Prostaglandin F2α ethanolamide	380.3	60	344.3/283.3	17/20
		**Nucleobases and Nucleosides**		
**Analyte**	**Precursor Ion (*m*/*z*)**	**Declustering Potential (V)**	**Product Ions (quant/qual, *m*/*z*)**	**CE (quant/qual, eV)**
Cytosine	112.1	70	95.0/69.0	25/25
Uracil	113.0	40	96.0/70.0	23/25
Thymine	127.0	70	110.0/84.0	20/25
Hypoxanthine	137.0	110	110.0/119.0	29/30
Xanthine	153.0	60	110.1/136.0	28/25
2'-Deoxycytidine	228.0	40	112.0/95.0	25/55
2'-Deoxythymidine	243.0	40	127.0/110.0	25/45
Uridine	245.1	50	113.0/96.0	20/55
2'-Deoxyadenosine	252.1	60	136.0/119.1	20/65
2'-Deoxyguanosine	268.1	50	152.0/135.0	30/50
Inosine	269.0	55	137.0/110.0	25/55
Guanosine	284.0	70	152.0/135.0	35/55
		**Others**		
**Analyte**	**Precursor Ion (*m*/*z*)**	**Declustering Potential (V)**	**Product Ions (quant/qual, *m*/*z*)**	**CE (quant/qual, eV)**
Acetamide	60.0	60	43.0/44.0	25/50
Urea	61.0	50	44.0/61.0	30/8
		**Internal Standards**		

**Analyte**	**Precursor ion (*m*/*z*)**	**Declustering Potential (V)**	**Product Ions (quant/qual, *m*/*z*)**	**CE (quant/qual, eV)**
IS Glycine	212.1	70	77.1/136.1	15/25
IS Alanine	228.1	70	93.1/136.1	15/30
IS Serine	244.1	70	109.1/136.1	15/30
IS DL-Proline	258.1	70	123.1/76.0	18/35
IS Threonine	260.1	70	125.1/79.1	15/30
IS Valine	260.1	70	125.1/79.1	15/25
IS L-Asparagine	272.1	70	255.1/90.1	15/25
IS L-Aspartic acid	272.1	70	136.1/91.1	30/25
IS Isoleucine	276.1	70	77.1/95.1	40/20
IS Leucine	276.1	70	48.1/141.1	60/15
IS L-Glutamic acid	288.1	70	153.1/88.1	15/35
IS L-Glutamine	288.1	70	135.1/89.1	25/38
IS Methionine	292.1	70	157.1/140.1	15/25
IS L-Histidine	296.1	70	203.1/115.1	15/30
IS DL-Phenylalanine	308.1	70	127.1/173.1	25/15
IS L-Arginine	320.1	70	183.1/226.1	25/15
IS Tyrosine	324.1	70	189.1/143.1	15/30
IS Tryptophan	348.1	70	195.1/151.1	25/35
IS Putrescin	363.1	70	270.1/118.1	15/35
IS Lysine	427.1	70	334.1/198.1	20/25

*quant, quantifier; qual, qualifier; CE = collision energy; IS, internal standard.

Table S4. Selected reaction monitoring (SRM) transitions for liquid chromatography - tandem mass spectrometric measurements in negative ionization mode for non-lipid compounds.*

Negative Ionization Mode				
**Bile Acids**				
**Analyte**	**Precursor Ion (*m/z*)**	**Declustering Potential (V)**	**Product Pons (quant/qual, *m/z*)**	**CE (quant/qual, eV)**
Lithocholic acid	375.3	-140	375.3/375.3	-40/-8
Chenodeoxycholic acid	391.3	-150	391.3/373.3	-8/-50
Deoxycholic acid	391.3	-150	391.3/345.3	-8/-50
Hyodeoxycholic acid	391.3	-150	391.3/391.3	-8/-30
Usodeoxycholic acid	391.3	-150	391.3/373.3	-8/-50
Cholic acid	407.3	-180	407.3/343.3	-8/-50
Glycolithocholic acid	432.3	-150	432.3/74.0	-10/-65
Glycochenodeoxyxcholic acid	448.3	-150	448.3/74.0	-8/-80
Glycodeoxycholic acid	448.3	-150	448.3/74.0	-8/-80
Glycohyodeoxycholic acid	448.3	-150	448.3/74.0	-8/-80
Glycoursodeoxycholic acid	448.3	-150	448.3/74.0	-8/-80
Glycocholic acid	464.3	-150	464.3/74.0	-8/-90
Taurolithocholic acid	482.3	-150	482.3/80.0	-8/-120
Taurochenodeoxycholic acid	498.3	-150	498.3/80.0	-10/-115
Taurodeoxycholic acid	498.3	-150	498.3/80.0	-10/-115
Taurohyodeoxycholic acid	498.3	-150	498.3/80.0	-10/-115
Tauroursodeoxycholic acid	498.3	-150	498.3/80.0	-10/-115
Taurocholic acid	514.3	-200	514.3/80.0	-8/-120
Tauromurocholic acid	514.3	-200	514.3/80.0	-8/-120
Taurolithocholic acid 3-sulfate	562.3	-150	482.3/562.3	-70/-10
**Carboxylic Acids**				
**Analyte**	**Precursor Ion (*m/z*)**	**Declustering Potential (V)**	**Product Ions (quant/qual, *m/z*)**	**CE (quant/qual, eV)**
Lactic acid	89.0	-50	43.0/41.0	-18/-40
3-Hydroxyglutaric acid	147.0	-50	85.0/43.0	-15/-35
cis-Aconitic acid	173.0	-50	85.0/129.0	-23/-15
Abscisic acid	263.1	-80	153.1/219.1	-18/-20
12-[[(Cyclohexylamino)carbonyl]amino]-dodecanoic acid	339.3	-60	214.1/240.0	-30/-25

Fatty Acids				
**Analyte**	**Precursor Ion (*m*/*z*)**	**Declustering Potential (V)**	**Product Ions (quant/qual, *m*/*z*)**	**CE (quant/qual, eV)**
Decanoic acid	171.1	-60	171.1/171.1	-8/-30
Undecanoic acid	185.2	-80	185.2/185.2	-8/-30
10-Hydroxydecanoic acid	187.1	-65	187.1/141.1	-8/-25
Lauric acid	199.2	-80	199.2/199.2	-8/-30
Tridecanoic acid	213.2	-70	213.2/213.2	-8/-30
Myristic acid	227.2	-95	227.2/227.2	-8/-30
Dodecanedioic acid	229.1	-60	211.1/167.0	-25/-28
14-Pentadecenoic acid	239.2	-80	239.2/239.2	-8/-30
Pentadecanoic acid	241.2	-60	197.1/197.1	-8/-30
Palmitoleic acid	253.2	-90	253.2/253.2	-8/-30
Palmitic acid	255.2	-100	255.2/255.2	-8/-30
Tetradecanedioic acid	257.2	-60	239.0/195.0	-26/-30
cis-10-Heptadecenoic acid	267.2	-90	267.2/267.2	-8/-30
Alpha-linolenic acid	277.2	-100	277.2/277.2	-8/-30
Gamma-linolenic acid	277.2	-100	277.2/277.2	-8/-30
Linoleic acid	279.2	-100	279.2/279.2	-8/-30
Elaidic acid	281.3	-110	281.3/281.3	-8/-30
Oleic acid	281.3	-110	281.3/281.3	-8/-30
Stearic acid	283.3	-110	283.3/283.3	-8/-30
Eicosapentaenoic acid	301.2	-70	301.2/257.2	-8/-20
Arachidonic acid	303.2	-90	303.2/259.2	-8/-20
11(Z),14(Z),17(Z)-Eicosatrienoic acid	305.3	-100	305.3/305.3	-8/-30
11(Z),14(Z)-Eicosadienoic acid	307.3	-100	307.3/307.3	-8/-30
cis-11-Eicosenoic acid	309.3	-110	309.3/309.3	-8/-30
cis-4,7,10,13,16,19-docosahexaenoic acid	327.2	-90	327.2/283.2	-8/-18
13(Z),16(Z)-Docosadienoic acid	335.3	-120	335.3/335.3	-30/-8
13(Z)-Docosenoic acid	337.3	-110	337.3/337.3	-8/-30
Behenic acid	339.3	-100	163.1/339.3	-43/-8
Arachidic acid	357.3	-40	311.3/357.3	-20/-8
Nervonic acid	365.3	-120	365.3/365.3	-30/-8
**Internal Standards**				
**Analyte**	**Precursor Ion (*m*/*z*)**	**Declustering Potential (V)**	**Product Ions (quant/qual, *m*/*z*)**	**CE (quant/qual, eV)**
IS Lithocholic acid	379.3	-140	379.0/379.3	-40/-10
IS Chenodeoxycholic acid	395.3	-150	395.0/377.0	-8/-50
IS Cholic acid	412.3	-180	412.0/348.0	-8/-50

IS Taurochenodeoxycholic acid	502.3	-150	502.0/80.0	-10/-115
IS Taurocholic acid	518.3	-200	518.0/80.0	-8/-120

*quant = quantifier, qual = qualifier, CE = collision energy.

Table S5. Selected reaction monitoring (SRM) transitions for liquid chromatography - tandem mass spectrometric measurements in positive ionization mode for lipids.*

**Positive Ionization Mode, Lipids**				
**Acylcarnitines**				
**Analyte**	**Precursor Ion (*m*/*z*)**	**Declustering Potential (V)**	**Product Ions (quant/qual, *m*/*z*)**	**CE (quant/qual, eV)**
Acetylcarnitine	204.1	50	204.1/85.1	5/27
Propionylcarnitine	218.1	50	159.0/85.1	20/29
Butyrylcarnitine	232.1	50	85.1/173.1	29/20
Tiglylcarnitine	244.2	50	85.1/185.1	31/20
Valerylcarnitine	246.2	50	85.1/187.1	29/20
Hexanoylcarnitine	260.2	50	85.1/201.2	33/20
Glutarylcarnitine	276.2	61	85.1/217.1	35/20
2-trans-Octenoylcarnitine	286.2	70	227.1/85.1	20/35
Octanoylcarnitine	288.2	70	85.1/229.1	33/20
Adipoylcarnitine	290.2	70	85.1/231.1	37/20
2-trans-Decenoylcarnitine	314.2	70	85.1/255.1	38/20
Decanoylcarnitine	316.2	70	85.1/257.1	37/20
2-trans-Dodecenoylcarnitine	342.3	80	85.1/283.2	42/25
2-trans-Tetradecenoylcarnitine	370.3	80	311.2/85.1	25/47
2-trans-Hexadecenoylcarnitine	398.3	90	85.1/339.2	49/25
Palmitoylcarnitine	400.3	90	85.1/341.3	50/28
9,12-cis,cis-Octadecadienoylcarnitine	424.3	90	85.1/144.2	52/38
Oleoylcarnitine	426.4	100	85.1/367.3	55/25
Stearoylcarnitine	428.4	100	85.1/369.3	55/30
**Cholesterol Esters**				
**Analyte**	**Precursor Ion (*m*/*z*)**	**Declustering Potential (V)**	**Product Ions (quant/qual, *m*/*z*)**	**CE (quant/qual, eV)**
Cholesteryl myristate	614.6	200	369.3/161.1	23/55
Cholesteryl palmitoleate	640.6	200	369.3/161.1	27/55
Cholesteryl palmitate	642.6	200	369.3/161.1	27/55
Cholesteryl heptadecanoate	656.6	200	369.3/161.1	24/55
Cholesteryl linoleate	666.6	220	369.3/161.1	25/40
Cholesteryl oleate	668.6	220	369.3/161.1	25/60
Cholesteryl stearate	670.6	220	369.3/161.1	25/55
Cholesteryl eicosapentaenoate	688.6	100	369.3/161.1	25/60
Cholesteryl arachidonate	690.6	220	369.3/161.1	30/45
Cholesteryl arachidate	698.7	100	369.3/161.1	25/55
Cholesteryl docosapentaenoate	716.6	220	369.3/161.1	30/50

Ethanolamides				
**Analyte**	**Precursor ion (*m*/*z*)**	**Declustering potential (V)**	**Product ions (quant/qual, *m*/*z*)**	**CE (quant/qual, eV)**
Palmitoleoyl ethanolamide	298.3	70	62.0/281.2	30/22
Palmitoyl ethanolamide	300.3	65	62.0/57.0	33/50
Linoleoyl ethanolamide	324.3	70	62.0/44.0	35/65
Oleoyl ethanolamide	326.3	70	62.0/44.0	38/65
Stearoyl ethanolamide	328.3	60	62.0/57.0	33/50
Eicosapentaenoyl ethanolamide	346.3	60	91.0/62.0	65/30
Arachidonoyl ethanolamide	348.3	60	62.0/91.0	35/70
Docosahexaenoyl ethanolamide	372.4	70	91.0/62.0	70/40
Docosatetraenoyl ethanolamide	376.3	70	62.0/91.0	40/70
**Glycerides**				
**Analyte**	**Precursor Ion (*m*/*z*)**	**Declustering Potential (V)**	**Product Ions (quant/qual, *m*/*z*)**	**CE (quant/qual, eV)**
2-Linoleoyl glycerol	372.3	50	263.2/337.3	15/20
2-Arachidonoyl glycerol	379.3	80	287.2/269.1	20/25
1,2-Dipalmitoyl-sn-glycerol	586.5	80	551.5/313.3	27/28
1,2-Dioleoyl-sn-glycerol	638.5	100	339.3/603.6	37/35
1,3-Dipalmitoyl-2-oleoylglycerol	850.8	100	577.4/551.4	45/35
1,2-Dilinoleoyl-3-palmitoylglycerol	872.8	120	599.5/575.5	42/40
1,3-Dioleyl-2-palmitoylglycerol	876.8	100	603.6/577.5	40/40
**Phospholipids**				
**Analyte**	**Precursor Ion (*m*/*z*)**	**Declustering Potential (V)**	**Product Ions (quant/qual, *m*/*z*)**	**CE (quant/qual, eV)**
1-Myristoyl-2-hydroxy-sn-glycero-3-phosphatidylcholine	468.3	100	184.1/104.0	35/32
1-Palmitoyl-2-hydroxy-sn-glycero-3-phosphatidylcholine	496.3	100	184.1/104.0	35/35
1-Heptadecanoyl-2-hydroxy-sn-glycero-3-phosphatidylcholine	510.4	120	184.0/125.1	40/65
1-Oleoyl-2-hydroxy-sn-glycero-3-phosphatidylcholine	522.4	80	184.1/104.0	40/38
1-Stearoyl-2-hydroxy-sn-glycero-3-phosphatidylcholine	524.4	80	184.1/104.0	38/35
C16 Sphingomyelin	703.6	100	184.1/86.0	40/80
1-Palmitoyl-2-myristoyl-sn-glycero-3-phosphatidylcholine	706.5	130	184.1/125.1	45/90

1-1(Z)-Hexadecenyl-2-palmitoyl-sn-glycero-3-phosphatidylcholine	718.6	140	184.0/125.1	40/85
1,2-Dipalmitelaidoyl-sn-glycero-3-phosphatidylcholine	730.5	140	184.0/125.1	40/85
1-O-Hexadecyl-2-arachidonoyl-sn-glycero-3-phosphatidylcholine	768.6	130	184.1/125.1	45/90
1,2-Dielaidoyl-sn-glycero-3-phosphatidylcholine	786.6	140	184.1/125.1	45/90
1-Oleoyl-2-stearoyl-sn-glycero-3-phosphatidylcholine	788.6	140	184.1/125.1	45/90
1,2-Distearoyl-sn-glycero-3-phosphatidylcholine	790.6	90	184.1/125.1	45/90
1-Stearoyl-2-arachidonoyl-sn-glycero-3-phosphatidylcholine	810.6	130	184.1/125.1	45/90
		**Sterols**		
**Analyte**	**Precursor Ion (*m*/*z*)**	**Declustering Potential (V)**	**Product Ions (quant/qual, *m*/*z*)**	**CE (quant/qual, eV)**
Cortexolone	347.2	75	347.2/109.1	8/50
24-Dehydrocholesterol	367.3	90	367.3/69.0	10/55
25-Hydroxycholesterol	367.4	90	367.3/367.4	20/5
Cholesterol	369.4	90	369.4/369.4	8/30
Dehydroergosterol	377.3	80	377.3/157.0	5/45
5,6α-Epoxycholesterol	385.4	90	367.3/159.0	20/40
5α,6β-Dihydroxycholestanol	385.4	90	367.3/385.4	20/8
25(R)-27-Hydroxycholesterol	385.4	80	161.1/69.0	30/55
Cholecalciferol	385.4	90	367.3/259.2	20/20
Doxercalciferol	395.3	80	395.3/377.3	20/20
Stigmasterol	395.3	90	395.3/69.0	5/45
β-Sitosterol	397.4	90	397.4/69.0	5/40
Calciferol	397.4	80	397.4/379.4	8/20
7-Ketocholesterol	401.3	90	95.0/81.0	55/60

*quant, quantifier; qual, qualifier; CE, collision energy.

Table S6. Compounds measured by anion exchange chromatography coupled to high resolution mass spectrometry (AIC-HR-MS).

Compound class	Analyte	[M-H]-
Carboxylic acids	Acetic acid	59.0139
	Propionic acid	73.0295
	Glycolic acid	75.0088
	Pyruvic acid	87.0088
	Butyric acid	87.0452
	Isobutyric acid	87.0452
	Lactic acid	89.0244
	Acetoacetic acid	101.0244
	Isovaleric acid	101.0608
	2-Methylbutyric acid	101.0608
	Pivalic acid	101.0608
	Valeric acid	101.0608
	Malonic acid	103.0037
	2-Hydroxybutyric acid	103.0401
	3-Hydroxybutyric acid	103.0401
	3-Hydroxyisobutyric acid	103.0401
	Glyceric acid	105.0193
	Fumaric acid	115.0037
	2-Ketoisovaleric acid	115.0401
	3,3-Dimethylbutyric acid	115.0765
	2,2-Dimethylbutyric acid	115.0765
	2-Ethylbutyric acid	115.0765
	Hexanoic acid	115.0765
	2-Methylvaleric acid	115.0765
	3-Methylvaleric acid	115.0765
	4-Methylvaleric acid	115.0765
	Methylmalonic acid	117.0193
	Succinic acid	117.0193
	Benzoic acid	121.0295
	Ketoisoleucine	129.0557
	Malic acid	133.0142
	Phenylacetic acid	135.0452
	4-Hydroxybenzoic acid	137.0244
	Caprylic acid	143.1078
	α-Ketoglutaric acid	145.0142
	3-Hydroxyglutaric acid	147.0299
	3-Phenylpropionic acid	149.0608
	3-Hydroxyphenylacetic acid	151.0401

	4-Hydroxyphenylacetic acid	151.0401
	Phenylpyruvic acid	163.0401
	3-(3-Hydroxyphenyl)propionic acid	165.0557
	cis-Aconitic acid	173.0092
	2-Hydroxy-3-isopropylbutanedioic acid	175.0612
	10-Hydroxydecanoic acid	187.1340
	Citric acid	191.0197
	Isocitric acid	191.0197
	trans-Ferulic acid	193.0506
Sugar phosphates	Ribose 5-phosphate	229.0119
	Glucose 1-phosphate	259.0224
	Fructose 6-phosphate	259.0224
	Galactose 1-phosphate	259.0224
	Glucose 6-phosphate	259.0224
	Mannose 6-phosphate	259.0224
	Sedoheptulose 7-phosphate	289.0330
	Fructose 1,6-bisphosphate	338.9888
Sugar related	Phosphoenolpyruvic acid	166.9751
	Glyceraldehyde 3-phosphate	168.9908
	3-Phosphoglyceric acid	184.9857
	Galacturonic acid	193.0354
	N-Acetylglucosamine	220.0827
	6-Phosphogluconic acid	275.0174
	Sialyllactose	632.2044
Sugars	Pentoses	149.0455
	Hexoses	179.0561
	Sucrose	341.1089
Amino acid related	Pyroglutamic acid	128.0353
	N-(2-Hydroxyethyl)-iminodiacetic acid	176.0564
	N-Acetyltyrosine	222.0772
Others	Methyl phosphate	110.9853
	Uric acid	167.0211

Table S7. Differences in age-related development of selected plasma water-soluble metabolites (mg/L) in suckling and newly weaned piglets.*

		Day of Life (DoL)							*p*-value
**Metabolite**	**Metabolic Pathway**	**7**	**14**	**21**	**28**	**31**	**35**	**SEM**	**DoL**
Succinic acid	TCA cycle	1.43	0.61	0.43	0.49	0.46	0.44	0.17	<0.001
Fumaric acid	TCA cycle	0.27	0.14	0.08	0.10	0.10	0.12	0.02	<0.001
Citric acid	TCA cycle	42	44	36	37	29	35	2.58	0.004
Cis aconitic acid	TCA cycle	0.87	0.87	0.71	0.67	0.53	0.64	0.05	<0.001
Malic acid	TCA cycle	2.35	1.38	1.01	1.09	1.06	1.27	0.19	<0.001
Pyruvic acid	Glycolysis, gluconeogenesis	16	12	12	12	13	11	1.18	0.037
Lactic acid	Anaerobic ATP generation	261	192	174	180	157	173	18.74	0.003
Galactose-1-phosphate	Intraconversion of glucose and uridine diphosphate galactose	0.04	0.03	0.04	0.04	0.02	0.01	0.005	<0.001
Glyceric acid	Glycerol catabolism	0.14	0.12	0.10	0.09	0.08	0.08	0.01	<0.001
D-Sedoheptulose 7-phosphate	Pentose phosphate pathway	0.11	0.08	0.06	0.04	0	0	0.01	0.011
Acetic acid	Microbial metabolism	0.68	1.05	1.13	1.38	2.62	4.72	0.31	<0.001
Propionic acid	Microbial metabolism	0	0	0	0	0.24	0.33	0.07	0.336
Butyric acid	Microbial metabolism	0	0	0	0	0	0.86	0.11	0.650
Benzoic acid	Plant origin	0	0	0	0	0.39	0.63	0.16	0.025
Hippuric acid	Benzoid acid catabolism	1.83	1.89	2.25	1.89	12.86	21.39	1.70	<0.001
Uric acid	Purine catabolism	1.56	0.52	0.29	0	0.06	0	0.09	<0.001
Chenodeoxycholic acid	Bile acid	0.82	0.72	1.43	3.00	0.49	0.79	0.29	<0.001
Deoxycholic acid	Bile acid	0.08	0.06	0.06	0.06	0.07	0.06	0.01	0.555
Hyodeoxycholic acid	Bile acid	2.19	3.65	7.47	10.89	1.26	2.21	1.22	<0.001
Glycohyodeoxycholic acid	Bile acid	0.52	1.32	3.17	3.43	3.02	2.14	0.58	0.003
Glycochenodeoxycholic acid	Bile acid	0.64	0.71	0.86	1.45	1.30	0.93	0.15	0.004
Taurohyodeoxycholic acid	Bile acid	0.31	0.50	0.81	0.92	0.42	0.44	0.17	0.034
Taurochenodeoxycholic acid	Bile acid	0.41	0.25	0.29	0.48	0.47	0.30	0.06	0.037

Alanine	Proteinogenic AA	159	129	125	135	139	176	17.33	0.235
Arginine	Proteinogenic AA	19.6	32.2	18.4	14.3	7.8	8.0	2.21	<0.001
Asparagine	Proteinogenic AA	17.4	12.9	8.9	9.8	5.7	8.7	1.05	<0.001
Cysteine	Proteinogenic AA	4.4	5.6	5.1	5.0	5.4	4.2	0.42	0.092
Glutamine (log_{10})	Proteinogenic AA	2.1	2.1	2.0	2.2	2.0	2.2	0.04	<0.001
Glutamate	Proteinogenic AA	70	76	74	65	82	118	13.40	0.070
Glycine	Proteinogenic AA	63	73	64	64	55	42	4.64	0.001
Histidine (log_{10})	Proteinogenic AA	8.5	8.5	8.5	8.4	8.4	8.4	0.05	0.881
Isoleucine	Proteinogenic AA	46	42	28	32	38	32	3.75	0.004
Leucine	Proteinogenic AA	79	79	65	71	46	51	6.88	0.005
Lysine	Proteinogenic AA	40	45	35	38	32	40	6.30	0.702
Methionine	Proteinogenic AA	16	15	10	11	12	13	1.35	0.012
Phenylalanine	Proteinogenic AA	21	20	17	16	14	15	1.25	0.004
Proline (log_{10})	Proteinogenic AA	2.0	1.9	1.8	1.8	1.3	1.4	0.03	<0.001
Serine	Proteinogenic AA	34	40	25	24	15	14	2.26	<0.001
Threonine	Proteinogenic AA	40	51	28	15	36	50	4.01	<0.001
Tryptophan	Proteinogenic AA	8.3	14	12	13	8.5	9.5	0.87	<0.001
Tyrosine	Proteinogenic AA	48	47	35	38	15	18	3.08	<0.001
Valine	Proteinogenic AA	46	46	35	35	38	44	3.99	0.112
cis-4-Hydroxyproline	Proteinogenic AA	15	20	15	14	10	10	0.77	<0.001
Citrulline	Urea cycle	24	28	23	23	12	12	2.22	<0.001
Ornithine	Urea cycle	10.2	9.6	7.0	5.7	2.3	3.6	0.81	<0.001
Urea (log_{10})	Urea cycle	7.0	6.9	6.8	7.0	6.9	6.8	0.07	0.554
beta-Alanine	Precursor in carnosine synthesis; putative "small molecule neurotransmitter"	2.0	1.1	0.92	0.64	0.48	0.44	0.12	<0.001
Alpha-aminoadipic acid	Lysine metabolism	2.9	2.3	2.8	3.3	2.6	2.4	0.73	0.910
Alpha-aminobutyric acid	Catabolism of methionine and threonine	1.0	0.69	0.60	1.5	7.0	4.0	0.38	<0.001

Asymmetric dimethylarginine	Inhibitor in nitric oxide production; key chemical for normal endothelial function	0.28	0.42	0.31	0.31	0.24	0.22	0.04	0.017
Symmetric dimethylarginine	Marker for renal function	0.40	0.34	0.30	0.29	0.32	0.28	0.03	0.174
Homoarginine	Intermediate in production of nitric oxide	0.17	0.20	0.17	0.17	0.11	0.10	0.02	0.012
5-Aminopentanoic acid	Derivative from valeric acid; GABA antagonists	0.18	0.17	0.21	0.30	0.16	0.22	0.03	0.028
Cadaverin	Lysine catabolism	0.74	0.70	0.65	0.66	0.62	0.60	0.12	0.974
Carnosine	Histidine-beta-alanine catabolism; pyrimidine catabolism	3.1	3.8	4.0	4.7	4.1	4.0	0.34	0.052
Histamine	Neurotransmitter; inflammation; gut function regulator	0.29	0.14	0.22	0.06	0.24	0	0.12	0.538
Putrescine	Ornithine catabolism; essential factor for cell division	1.5	1.5	1.2	1.3	1.3	1.2	0.26	0.927
Sarcosine	Intermediate in the metabolism of choline to glycine	3.6	3.8	2.9	2.2	1.9	1.6	0.33	0.001
Spermidine	Membrane potential; control of intracellular pH and volume; autophagy; inflammation; lipid metabolism; regulation of cell growth, proliferation and death	0.55	0.52	0.42	0.46	0.39	0.38	0.06	0.217
Serotonin	Neurotransmitter	0.10	0.11	0.13	0.07	0.17	0.06	0.06	0.729
Creatine	Recycling of ATP, primarily in muscle and brain tissue; buffer	8.4	12.7	12.5	19.9	26.6	12.7	5.59	0.301
Creatinine (log_{10})	Muscle/protein metabolism: breakdown product of creatine phosphate	7.0	7.1	7.1	7.1	7.2	7.2	0.03	<0.001

Taurine	Conjugation of bile acids, antioxidation, osmoregulation, membrane stabilization, and modulation of calcium signaling	20	19	15	13	7.3	4.2	1.27	<0.001
Methionine sulfoxide	Product of methionine oxidation, radical	0.5	0.4	0.3	0.4	0.4	0.6	0.07	0.124
Betaine	Methylation reactions, detoxification of homocysteine	29	31	32	34	19	17	2.99	<0.001
Trimethylamine-N-oxide	Gut microbial metabolism	1.6	1.7	1.0	0.8	1.9	2.5	0.31	0.002
Choline	Methylation reactions, detoxification of homocysteine; bile acids	2.4	1.6	1.2	1.1	1.1	0.8	0.15	<0.001
Homocysteine	Methylation reactions; conversion to cysteine	0.64	1.1	1.0	1.1	1.1	1.0	0.22	0.737
Hypoxanthine	Purine derivative	5.3	4.1	2.7	2.0	2.0	1.4	0.32	<0.001
Phenylacetylglycine	Phospholipid catabolism	1.2	1.6	1.9	2.2	3.1	2.6	0.31	0.005
Ethanolamine	Cellular membrane synthesis	0.5	0.6	0.4	0.2	0.2	0.2	0.06	<0.001
1-Methyl-histidine	Porcine vasoactive intestinal polypeptide-1	1.7	1.2	1.2	1.3	1.5	1.7	0.17	0.092
2-Methyl-histidine	Porcine vasoactive intestinal polypeptide-1	1.5	1.2	1.1	1.3	1.4	1.6	0.13	0.080
3-Methyl-histidine	Porcine vasoactive intestinal polypeptide-1	1.2	1.1	1.0	1.1	1.1	1.0	0.13	0.857
Thyroxine	Thyroid metabolism; basal metabolism	0.08	0.09	0.08	0.09	0.09	0.09	0.01	0.926
Kynurenine	Niacin/Tryptophan metabolism; involved in dilating blood vessels during inflammation and regulating immune responses	0.18	0.20	0.24	0.19	0.12	0.18	0.04	0.324
Ketoisoleucine	AA intermediate	1.6	2.2	1.9	1.9	2.8	2.0	0.19	<0.001
3-OH glutaric acid	Catabolism of AA; human marker for glutaric aciduria type I	0.41	0.33	0.26	0.33	0.34	0.45	0.04	0.008
N-(2 Hydroxyethyl) iminodiacetic acid	Glycine metabolism	0.02	0.01	0.01	0	0	0	0.002	<0.001
N-acetyltyrosine	Tyrosine metabolism, urinary excretion	0.07	0.06	0.04	0	0	0	0.01	0.008
Pyroglutamate	Natural AA derivative (little studied)	0.85	0.96	1.00	1.03	1.21	1.38	0.08	<0.001

2-Hydroxybutyric acid	Ketone, threonine catabolism; synthesis of glutathione; insulin resistance marker	1.06	0.74	0.70	1.07	3.72	2.05	0.32	<0.001
2-Keto isovaleric acid	Ketone, valine metabolism, precursor to pantothenic acid	0.18	0.25	0.21	0.18	0.25	0.20	0.02	0.009

*Values are least squares means ± standard error of the mean (SEM). Piglets were weaned on day 28 of life. AA, amino acid; GABA, gamma-aminobutyric acid; TCA, tricarbon cycle.

Table S8. Differences in age-related development of selected plasma lipids (mg/L) in suckling and newly weaned piglets receiving only sow milk or additionally creep feed from day of life 10 during the suckling phase.*

Day of Life (DoL)	7	14		21		28		31		35			*p*-value		
Feeding (Feed)	Sow Milk	Sow Milk	Creep Feed	Sow Milk	Creep Feed	Sow Milk	Creep Feed	Sow Milk	Creep Feed	Sow Milk	Creep Feed	SEM	DoL	Feed	DoL × Feed
C0 Acyl-carnitine	0.35	0.23	0.31	0.20	0.27	0.18	0.30	0.21	0.24	0.27	0.19	0.04	0.031	0.030	0.193
C2 Acyl-carnitine	1.43	0.75	1.06	0.56	0.78	0.45	0.96	0.86	1.50	0.59	0.36	0.22	<0.001	0.038	0.368
C3 Acyl-carnitine	0.08	0.043	0.059	0.046	0.054	0.044	0.10	0.077	0.10	0.15	0.11	0.02	0.006	0.301	0.257
C9 Acyl-carnitine	0.02	0.015	0.016	0.027	0.026	0.029	0.039	0.026	0.032	0.030	0.028	0.003	<0.001	0.046	0.191
lysoPC a C18:0	11.48	14.5	12.5	14.4	13.8	12.2	11.2	8.8	9.0	10.0	6.5	0.98	<0.001	0.019	0.470
PC aa C30:2	0.03	0.06	0.04	0.08	0.07	0.07	0.05	0.05	0.05	0.10	0.01	0.01	0.006	0.001	0.052
PC aa C36:0	9.45	9.1	7.9	7.6	7.7	6.8	6.1	5.4	5.4	5.0	2.7	0.69	<0.001	0.042	0.486
PC aa C36:2	35.31	44	37	35	36	31	28	12.3	9.9	15.1	6.4	2.99	<0.001	0.025	0.674
PC aa C38:3	3.61	4.1	3.5	3.8	3.6	3.7	3.5	2.8	2.9	2.7	1.3	0.34	<0.001	0.045	0.299
PC ae C34:1	25.61	35.1	29.0	35.5	31.0	33.6	30.4	19.7	17.5	22.0	13.8	2.82	<0.001	0.008	0.815
PC ae C34:2	18.98	29.5	27.2	35.0	31.5	37.9	32.0	19.3	15.7	20.9	11.6	2.73	<0.001	0.006	0.676
PC ae C36:0	0.33	0.50	0.46	0.56	0.49	0.30	0.26	0.21	0.07	0.25	0.04	0.06	<0.001	0.023	0.360
PC ae C36:1	2.75	2.8	2.7	2.8	2.9	2.3	2.1	2.0	1.2	2.7	1.5	0.32	0.013	0.034	0.240
PC ae C36:3	4.43	5.6	4.7	6.1	5.1	6.0	5.8	4.1	3.4	4.4	2.9	0.53	<0.001	0.008	0.869
PC ae C38:2	0.59	0.70	0.62	0.63	0.63	0.47	0.34	0.23	0.08	0.32	0.08	0.07	<0.001	0.027	0.291
PC ae C40:5	1.34	1.4	1.2	1.5	1.2	1.4	1.3	1.4	1.3	1.3	0.73	0.16	0.164	0.025	0.737
PC ae C40:6	2.92	2.9	2.5	2.9	2.6	2.5	2.5	2.3	1.9	2.2	1.2	0.29	0.008	0.023	0.685
SM (OH) C14:1	1.85	1.5	1.2	1.5	1.2	1.2	0.93	0.82	0.71	1.1	0.27	0.23	0.001	0.018	0.592
SM C18:0	7.73	7.7	6.8	11.3	7.9	10.1	10.0	15.0	16.3	14.9	8.7	1.28	<0.001	0.029	0.040
SM C24:0	1.02	1.9	1.7	3.5	3.0	2.7	2.2	2.3	1.9	2.6	1.6	0.40	<0.001	0.042	0.876
SM C24:1	4.99	7.1	6.1	10.8	8.3	8.7	8.0	10.2	11.1	11.0	7.2	0.82	<0.001	0.027	0.016

*Values are least squares means ± standard error of the mean (SEM). Piglets were weaned on day 28 of life. lysoPC a, lysophosphatidylcholine with acyl residue C; PC aa C, phosphatidylcholine with diacyl residue sum C; PC ae C, phosphatidylcholine with acyl-alkyl residue sum C; SM C, sphingomyelin with acyl residue sum C; SM (OH) C, hydroxysphingomyelin with acyl residue sum C.

Table S9. Differences in age-related development of selected plasma triglycerides (mg/L) in suckling and newly weaned piglets receiving only sow milk or additionally creep feed from day of life 10 during the suckling phase.*

Day of Life (DoL)	7	14		21		28		31		35			*p*-value		
Feeding (Feed)	Sow Milk	Sow Milk	Creep Feed	Sow Milk	Creep Feed	Sow Milk	Creep Feed	Sow Milk	Creep Feed	Sow Milk	Creep Feed	SEM	DoL	Feed	DoL × Feed
TG 14:0_34:1	3.96	5.18	6.58	6.02	11.1	3.52	4.56	0	0	0	0	1.19	<0.001	0.084	0.134
TG 14:0_34:2	2.13	3.07	4.81	3.97	7.89	2.50	3.40	0.25	0	0.03	0	0.88	<0.001	0.041	0.089
TG 14:0_36:2	2.95	2.74	3.32	2.79	4.60	1.09	1.75	0	0	0	0	0.62	<0.001	0.240	0.452
TG 14:0_36:3	1.19	1.14	1.67	1.16	2.81	0.73	1.29	0.25	0	0.01	0	0.39	<0.001	0.078	0.070
TG 16:0_32:0	14.85	20.4	25.8	27.1	32.3	17.9	20.1	1.39	0.60	2.80	0	2.89	<0.001	0.293	0.599
TG 16:0_32:1	10.51	16.2	22.5	22.2	43.0	16.2	19.3	2.19	0.83	1.83	0	4.43	<0.001	0.064	0.074
TG 16:0_32:2	2.18	3.46	5.83	5.19	10.7	3.90	4.97	0.86	0.22	0.47	0	1.18	<0.001	0.037	0.066
TG 16:0_33:1	0.16	0.06	0.26	0.28	0.89	0.27	0.32	0.03	0.01	0.05	0.01	0.13	0.001	0.132	0.032
TG 16:0_34:0	3.41	3.85	4.90	5.00	7.84	2.37	3.35	0	0	0	0	0.87	<0.001	0.203	0.286
TG 16:0_34:1	45.44	50.4	60.6	58.6	92.5	35.6	44.9	3.54	0	3.06	0	9.17	<0.001	0.188	0.182
TG 16:0_34:2	22.75	29.6	39.6	38.1	66.8	28.5	37.4	12.2	5.60	7.13	0.36	6.85	<0.001	0.101	0.083
TG 16:0_34:3	3.13	4.43	6.87	6.57	12.9	5.94	8.38	3.67	2.03	1.24	0	1.57	<0.001	0.070	0.096
TG 16:0_36:2	90.86	72.0	76.5	72.8	97.6	40.3	53.4	19.3	10.8	11.1	0	12.29	<0.001	0.814	0.514
TG 16:0_36:3	47.13	43.4	50.4	49.5	78.3	39.6	60.4	42.7	23.7	29.1	15.0	10.09	0.006	0.476	0.100
TG 16:0_36:4	6.80	5.96	8.77	8.60	16.8	14.9	20.8	26.2	16.4	22.8	17.6	4.05	0.013	0.802	0.181
TG 16:0_36:5	0.32	0.28	0.26	0.23	1.01	1.50	2.23	4.35	3.06	3.51	2.56	0.68	<0.001	0.807	0.506
TG 16:0_38:3	1.83	1.51	1.60	1.69	2.21	0.81	1.32	0	0	0.20	0	0.35	<0.001	0.884	0.571
TG 16:0_38:4	1.98	1.59	1.69	2.01	2.69	1.51	2.22	0.66	0.49	0.36	0	0.36	<0.001	0.484	0.433
TG 16:0_38:5	1.67	1.47	1.06	1.84	2.29	1.47	2.46	1.94	1.81	0.69	0.07	0.55	0.018	0.847	0.676
TG 16:0_38:6	0.34	0.24	0.19	0.41	0.62	0.62	1.20	1.28	0.95	0.45	0.25	0.28	0.020	0.743	0.592

TG 16:0_40:6	0.33	0.10	0.06	0.29	0.35	0.37	0.78	0.74	0.69	0.23	0.07	0.20	0.021	0.545	0.737
TG 16:1_30:1	1.99	2.68	4.74	3.99	8.84	3.26	3.78	0.81	0.77	1.14	0.50	0.90	<0.001	0.018	0.025
TG 16:1_32:0	10.40	17.2	24.6	24.8	49.8	21.3	23.0	2.81	2.01	3.74	0.03	5.30	<0.001	0.072	0.077
TG 16:1_32:1	10.65	17.8	26.8	24.3	50.1	19.0	22.7	5.01	3.93	4.33	0.34	5.22	<0.001	0.033	0.056
TG 16:1_32:2	2.62	3.63	6.84	5.66	11.70	4.51	5.83	1.21	0.80	0.92	0	1.24	<0.001	0.015	0.045
TG 16:1_33:1	0.80	0.45	0.95	0.85	1.67	1.16	1.67	0.66	0.70	0.41	0.61	0.26	0.002	0.057	0.216
TG 16:1_34:0	2.79	3.56	4.72	4.35	8.12	2.88	3.25	0.19	0	0.12	0	0.85	<0.001	0.077	0.103
TG 16:1_34:1	37.33	47.2	60.6	58.8	101.8	40.8	47.0	7.84	3.79	7.22	0	10.25	<0.001	0.116	0.112
TG 16:1_34:2	17.55	22.1	33.4	30.3	57.7	24.2	31.6	10.6	6.99	6.28	0.47	5.91	<0.001	0.031	0.058
TG 16:1_34:3	2.70	3.55	6.39	5.33	10.8	4.91	6.85	2.48	1.75	1.14	0	1.23	<0.001	0.015	0.067
TG 16:1_36:1	5.98	4.58	5.38	5.00	7.50	2.58	3.44	0.11	0	0.06	0	0.92	<0.001	0.341	0.436
TG 16:1_36:2	27.30	23.5	29.8	25.2	38.0	14.1	19.2	6.42	2.94	5.19	0	4.26	<0.001	0.247	0.201
TG 16:1_36:3	12.13	10.7	14.3	12.0	20.3	9.57	14.4	7.85	5.17	5.57	1.85	2.32	<0.001	0.071	0.064
TG 16:1_36:4	2.03	1.75	3.03	2.38	4.93	2.45	4.06	3.48	2.15	2.49	1.35	0.73	0.096	0.099	0.037
TG 16:1_38:3	0.26	0.17	0.31	0.26	0.52	0.08	0.13	0	0	0	0	0.08	<0.001	0.130	0.342
TG 16:1_38:5	0.16	0	0.04	0.17	0.29	0.18	0.49	0.50	0.45	0.15	0.06	0.13	0.009	0.234	0.602
TG 17:0_34:2	0.28	0.09	0.24	0.33	1.05	0.46	0.47	0.11	0	0.13	0	0.13	<0.001	0.126	0.013
TG 17:0_34:3	0.26	0.22	0.41	0.32	0.76	0.25	0.36	0.16	0	0.18	0	0.11	0.002	0.240	0.034
TG 17:0_36:3	0.22	0.06	0.16	0.16	0.29	0.15	0.31	0.31	0.03	0.42	0.21	0.10	0.440	0.977	0.073
TG 17:1_32:1	0.26	0.13	0.40	0.31	1.03	0.50	0.61	0.13	0.09	0.20	0.05	0.16	0.001	0.251	0.021
TG 17:1_34:1	0.99	0.69	1.03	1.12	1.91	0.81	1.04	0.19	0	0.15	0	0.25	<0.001	0.410	0.189
TG 17:1_34:2	0.37	0.26	0.55	0.56	1.23	0.66	0.81	0.44	0.06	0.44	0	0.18	0.001	0.474	0.014
TG 18:0_30:0	1.36	1.87	2.23	2.27	3.88	1.41	1.52	0	0	0	0	0.42	<0.001	0.143	0.226
TG 18:0_30:1	0.37	0.54	0.84	0.60	1.59	0.37	0.46	0	0	0.07	0	0.19	<0.001	0.028	0.050
TG 18:0_32:0	8.31	9.59	11.89	11.34	17.1	6.11	8.23	0	0	0.13	0	1.74	<0.001	0.163	0.270
TG 18:0_32:1	6.44	7.52	9.02	8.69	14.5	5.58	6.42	0.34	0	0.40	0	1.49	<0.001	0.203	0.155
TG 18:0_32:2	1.13	1.40	1.89	1.62	3.56	1.05	1.49	0	0	0	0	0.40	<0.001	0.055	0.058

TG 18:0_34:2	14.46	13.7	14.8	14.8	22.3	9.08	13.0	2.68	0.86	2.49	0	2.41	<0.001	0.346	0.222
TG 18:0_34:3	2.14	1.96	2.49	2.36	4.42	1.91	2.93	0.91	0.18	0.38	0	0.54	<0.001	0.144	0.080
TG 18:0_36:1	1.13	2.08	2.68	1.07	0.86	0.76	0.47	1.27	1.03	0.09	0.15	0.52	0.005	0.962	0.947
TG 18:0_36:2	13.49	7.44	7.88	6.54	8.75	3.35	5.75	2.99	2.36	3.01	1.56	1.48	<0.001	0.843	0.518
TG 18:0_36:3	11.92	6.65	7.20	6.67	9.51	5.16	8.33	7.91	5.55	7.11	5.56	1.76	0.033	0.869	0.406
TG 18:0_36:4	1.70	0.83	1.07	0.94	2.31	2.63	4.28	6.34	3.64	6.43	5.66	1.07	0.001	0.936	0.316
TG 18:1_30:0	14.36	18.2	21.9	20.7	36.4	12.6	15.3	0	0	1.19	0	3.52	<0.001	0.098	0.098
TG 18:1_30:1	5.72	6.95	9.19	7.89	15.1	5.40	6.58	0.49	0.14	0.90	0	1.48	<0.001	0.039	0.056
TG 18:1_30:2	0.48	0.50	0.76	0.62	1.64	0.27	0.54	0.04	0	0.06	0	0.19	<0.001	0.009	0.035
TG 18:1_32:0	73.60	83.2	97.6	98.2	148.5	56.9	71.8	0	0	3.07	0	14.79	<0.001	0.232	0.262
TG 18:1_32:1	60.73	70.5	87.7	80.5	137.1	52.9	61.7	7.38	2.10	7.99	0	13.69	<0.001	0.123	0.121
TG 18:1_32:2	10.44	10.8	14.9	12.6	22.7	9.01	11.1	2.86	1.45	2.15	0	2.31	<0.001	0.052	0.072
TG 18:1_32:3	0.59	0.48	0.78	0.59	1.09	0.26	0.47	0.33	0	0	0	0.19	0.002	0.301	0.232
TG 18:1_33:1	2.11	1.65	2.09	2.09	3.22	1.41	1.84	0.75	0.27	0.77	0.15	0.36	<0.001	0.420	0.119
TG 18:1_34:1	237.18	197.6	211.4	199.6	265.0	106.8	127.2	15.4	1.99	12.8	0	32.15	<0.001	0.833	0.590
TG 18:1_34:2	119.85	114.8	132.8	124.4	192.0	87.0	113.0	47.6	24.4	36.9	7.42	20.81	<0.001	0.340	0.170
TG 18:1_34:3	17.49	15.9	20.7	18.2	30.3	14.0	20.2	11.5	6.91	8.28	3.30	3.32	<0.001	0.110	0.063
TG 18:1_34:4	0.92	0.55	0.99	0.68	1.44	0.40	0.93	1.30	0.71	0.55	0.16	0.32	0.294	0.337	0.189
TG 18:1_35:2	2.04	1.38	1.50	1.39	2.00	1.16	1.32	0.83	0.33	1.12	0.32	0.30	0.001	0.705	0.192
TG 18:1_35:3	0.26	0.11	0.15	0.10	0.34	0.10	0.25	0.20	0.01	0.21	0.01	0.09	0.504	0.798	0.072
TG 18:1_36:1	25.89	15.0	15.3	13.4	16.1	5.95	9.84	4.11	3.10	4.12	1.63	2.75	<0.001	0.985	0.710
TG 18:1_36:2	151.10	90.2	98.1	84.3	99.3	39.4	53.3	35.9	25.5	39.2	22.2	15.82	<0.001	0.907	0.786
TG 18:1_36:3	97.23	65.5	73.2	64.0	86.2	42.5	64.8	60.2	38.3	63.7	46.7	13.86	0.047	0.815	0.406
TG 18:1_36:4	14.30	8.2	10.7	8.58	14.1	10.7	18.3	30.9	21.0	40.9	33.9	6.43	0.001	0.961	0.671
TG 18:1_36:5	1.24	0.35	0.56	0.32	0.93	0.94	1.94	6.28	4.19	8.05	6.65	1.28	<0.001	0.750	0.770
TG 18:1_38:5	2.85	1.24	1.22	1.71	1.97	1.59	3.11	2.91	3.01	1.74	1.17	0.56	0.006	0.516	0.512
TG 18:1_38:6	0.68	0.25	0.29	0.41	0.55	0.53	1.36	1.49	1.40	1.12	0.96	0.26	0.004	0.265	0.399

TG 18:2_28:0	0.38	0.74	1.37	1.09	3.01	0.71	1.09	0.08	0.04	0.24	0.04	0.31	<0.001	0.004	0.009
TG 18:2_30:0	4.32	6.49	8.91	8.15	15.95	6.16	7.76	0.68	0	0.78	0	1.58	<0.001	0.036	0.050
TG 18:2_30:1	1.99	2.56	4.16	3.44	6.96	2.48	3.38	0.39	0.05	0.28	0	0.69	<0.001	0.008	0.032
TG 18:2_32:0	18.94	25.6	32.4	32.9	59.7	25.8	35.1	8.40	2.96	6.96	0.91	6.01	<0.001	0.090	0.059
TG 18:2_32:1	15.37	19.8	27.4	25.1	47.0	21.2	28.7	9.05	5.33	4.93	0.05	4.69	<0.001	0.035	0.049
TG 18:2_32:2	2.93	3.27	5.24	4.56	8.88	4.43	6.11	2.71	1.40	2.00	0.60	0.97	<0.001	0.038	0.023
TG 18:2_33:0	0.03	0.04	0.10	0.18	0.46	0.14	0.26	0.02	0	0.07	0	0.08	<0.001	0.121	0.226
TG 18:2_33:1	0.39	0.23	0.43	0.50	1.03	0.51	0.71	0.26	0.02	0.18	0	0.15	<0.001	0.398	0.081
TG 18:2_34:0	5.91	5.79	6.60	6.41	10.58	5.03	7.03	3.25	1.87	2.45	0.73	1.22	<0.001	0.326	0.122
TG 18:2_34:1	65.00	66.3	75.2	72.2	113.9	58.6	80.7	41.4	24.1	30.2	12.8	13.00	<0.001	0.353	0.140
TG 18:2_34:2	23.18	22.0	31.6	26.5	50.4	32.3	47.1	41.5	24.8	36.7	26.2	7.40	0.200	0.287	0.046
TG 18:2_34:3	3.38	3.28	5.46	4.51	8.82	5.74	8.88	7.95	4.89	6.80	4.50	1.45	0.105	0.217	0.049
TG 18:2_35:1	0.34	0.28	0.37	0.32	0.58	0.29	0.37	0.22	0	0.42	0.09	0.12	0.190	0.670	0.116
TG 18:2_35:2	0.32	0.22	0.21	0.23	0.52	0.40	0.63	0.65	0.28	0.74	0.37	0.15	0.434	0.719	0.091
TG 18:2_36:0	0.70	0.27	0.40	0.30	0.53	0.14	0.35	0.27	0.07	0.25	0.02	0.14	0.015	0.983	0.331
TG 18:2_36:1	12.36	7.19	7.58	7.25	10.1	5.45	8.84	7.02	4.90	6.60	4.74	1.72	0.009	0.768	0.403
TG 18:2_36:2	46.98	30.6	35.3	30.3	43.2	22.7	35.7	37.2	22.9	38.6	31.1	7.45	0.268	0.719	0.320
TG 18:2_36:3	14.93	7.23	11.1	8.63	17.1	17.6	25.9	42.3	27.5	58.9	47.2	9.74	0.001	0.902	0.706
TG 18:2_36:4	0	0	0	0	0	9.64	14.2	29.9	19.1	48.1	40.3	8.98	<0.001	0.790	0.923
TG 18:2_36:5	0	0	0	0	0	1.00	1.40	5.63	3.57	9.42	7.50	1.74	<0.001	0.583	0.947
TG 18:2_38:4	0.52	0.10	0.18	0.39	0.61	0.61	1.14	1.05	0.84	1.08	0.86	0.21	0.010	0.425	0.410
TG 18:2_38:5	0.47	0.08	0.18	0.31	0.40	0.79	1.52	1.94	1.62	1.15	0.93	0.29	<0.001	0.569	0.496
TG 18:2_38:6	0	0	0	0	0	0.22	0.79	1.21	0.87	0.99	1.08	0.21	<0.001	0.564	0.427
TG 18:3_30:0	0.05	0.08	0.25	0.13	0.51	0.09	0.27	0.05	0	0	0	0.07	<0.001	0.006	0.030
TG 18:3_32:0	1.03	1.39	1.82	1.70	3.39	1.38	2.15	0.67	0.29	0.33	0	0.42	<0.001	0.099	0.097
TG 18:3_32:1	0.92	1.06	1.71	1.27	2.72	1.20	1.81	1.28	1.07	0.31	0	0.42	0.001	0.072	0.239
TG 18:3_34:0	0.13	0.02	0.10	0.01	0.19	0.05	0.20	0.37	0.15	0.09	0	0.11	0.353	0.645	0.355

TG 18:3_34:1	4.82	3.92	4.60	4.36	6.91	3.91	5.98	6.89	4.98	4.01	2.87	1.18	0.271	0.430	0.313
TG 18:3_34:2	1.65	1.14	1.78	1.47	3.04	2.41	3.84	6.46	4.23	4.26	3.51	0.95	0.008	0.637	0.266
TG 18:3_34:3	0.05	0	0.07	0	0.20	0.16	0.40	1.00	0.64	0.53	0.36	0.18	0.002	0.772	0.441
TG 18:3_36:1	0.42	0.11	0.18	0.12	0.34	0.07	0.42	0.79	0.32	0.57	0.51	0.16	0.063	0.842	0.144
TG 18:3_36:2	3.26	1.66	1.89	1.57	2.58	1.16	2.23	6.00	4.48	5.60	5.20	0.86	<0.001	0.812	0.627
TG 18: 3_36:3	1.03	0.04	0.40	0.10	0.61	1.08	1.96	6.48	4.49	8.03	6.98	1.28	<0.001	0.822	0.840
TG 18:3_36:4	0	0	0	0	0	0.44	0.70	3.88	2.46	5.51	4.50	0.99	<0.001	0.576	0.916
TG 20:1_32:1	0.24	0.30	0.39	0.34	0.68	0.13	0.11	0	0	0	0	0.10	<0.001	0.376	0.278
TG 20:1_34:2	0.70	0.57	0.75	0.64	1.04	0.28	0.45	0	0	0.07	0	0.16	<0.001	0.395	0.421
TG 20:2_32:0	0.40	0.50	0.57	0.63	1.03	0.26	0.31	0	0	0	0	0.14	<0.001	0.344	0.571
TG 20:2_32:1	0.29	0.29	0.58	0.52	1.12	0.22	0.30	0	0	0	0	0.15	<0.001	0.088	0.165
TG 20:2_34:1	1.52	1.05	1.22	1.23	1.71	0.67	0.78	0	0	0	0	0.30	<0.001	0.986	0.585
TG 20:3_34:1	2.88	2.53	2.48	3.28	3.84	2.04	3.04	0.65	0.68	0.64	0	0.45	<0.001	0.728	0.378
TG 20:3_34:2	0.87	0.73	0.92	1.57	1.88	1.03	2.05	0.99	0.94	0.55	0.20	0.29	<0.001	0.314	0.212
TG 20:3_36:3	0.97	0.77	0.66	1.02	0.93	0.84	1.65	1.04	1.24	1.10	1.07	0.23	0.483	0.252	0.299
TG 20:3_36:4	0.10	0.04	0.05	0.17	0.26	0.23	0.71	0.40	0.43	0.45	0.41	0.12	0.020	0.121	0.277
TG 20:4_32:0	2.26	2.93	2.89	4.06	4.61	2.48	3.84	0.32	0.10	0.30	0	0.57	<0.001	0.505	0.532
TG 20:4_32:1	1.74	2.20	2.32	3.13	3.85	2.17	3.61	1.53	1.32	0.62	0	0.57	<0.001	0.330	0.487
TG 20:4_32:2	0.03	0.01	0.11	0.13	0.38	0.12	0.36	0.35	0.24	0.06	0.02	0.12	0.075	0.224	0.591
TG 20:4_34:0	0.30	0.22	0.17	0.38	0.48	0.18	0.37	0.15	0.18	0.07	0	0.11	0.016	0.653	0.747
TG 20:4_34:1	8.01	6.67	6.43	8.38	8.94	6.35	9.26	4.96	5.58	2.62	0.77	1.48	<0.001	0.728	0.692
TG 20:4_34:2	3.56	3.41	3.38	4.83	5.56	4.70	7.44	6.14	5.98	3.21	1.76	1.16	0.005	0.446	0.546
TG 20:4_34:3	0.39	0.25	0.39	0.54	0.81	0.72	1.60	1.31	1.32	0.63	0.37	0.29	0.007	0.176	0.452
TG 20:4_36:2	5.05	2.56	2.44	3.04	2.83	2.81	5.41	5.86	5.55	3.25	3.08	0.91	0.003	0.504	0.531
TG 20:4_36:3	2.70	1.69	1.33	2.24	1.98	2.85	5.16	5.87	5.51	3.95	3.86	0.81	0.001	0.460	0.461
TG 20:4_36:4	0.14	0.02	0	0.17	0.21	0.91	1.96	3.28	3.17	2.45	2.38	0.48	<0.001	0.450	0.777
TG 20:4_36:5	0.02	0	0	0.02	0.05	0.20	0.50	0.71	0.87	0.41	0.30	0.14	<0.001	0.312	0.620

TG 20:5_34:1	0.33	0.27	0.18	0.39	0.44	0.42	0.47	0.54	0.36	0.19	0	0.18	0.239	0.582	0.956
TG 20:5_34:2	0	0	0	0.02	0.03	0.24	0.32	0.63	0.37	0.28	0.13	0.17	0.056	0.590	0.888
TG 20:5_36:2	0.13	0	0	0	0	0.06	0.11	0.47	0.34	0.33	0.29	0.09	<0.001	0.627	0.948
TG 22:5_32:0	0.47	0.33	0.52	0.57	0.49	0.57	0.64	0.86	0.49	0.55	0.61	0.09	0.261	0.403	0.039
TG 22:5_34:1	0.29	0.05	0.09	0.20	0.30	0.29	0.56	0.69	0.48	0.18	0.11	0.18	0.085	0.756	0.822
TG 22:5_34:2	0.09	0	0.02	0.02	0.32	0.39	0.76	0.93	0.60	0.43	0.29	0.17	0.002	0.408	0.283
TG 22:6_34:1	0.49	0.09	0.08	0.27	0.39	0.35	0.67	0.76	0.59	0.58	0.38	0.17	0.053	0.727	0.597
TG 22:6_34:2	0	0	0	0	0.02	0.18	0.42	0.96	0.54	0.72	0.60	0.16	<0.001	0.704	0.398

*Values are least squares means ± standard error of the mean (SEM). Piglets were weaned on day 28 of life. TG, triglyceride.

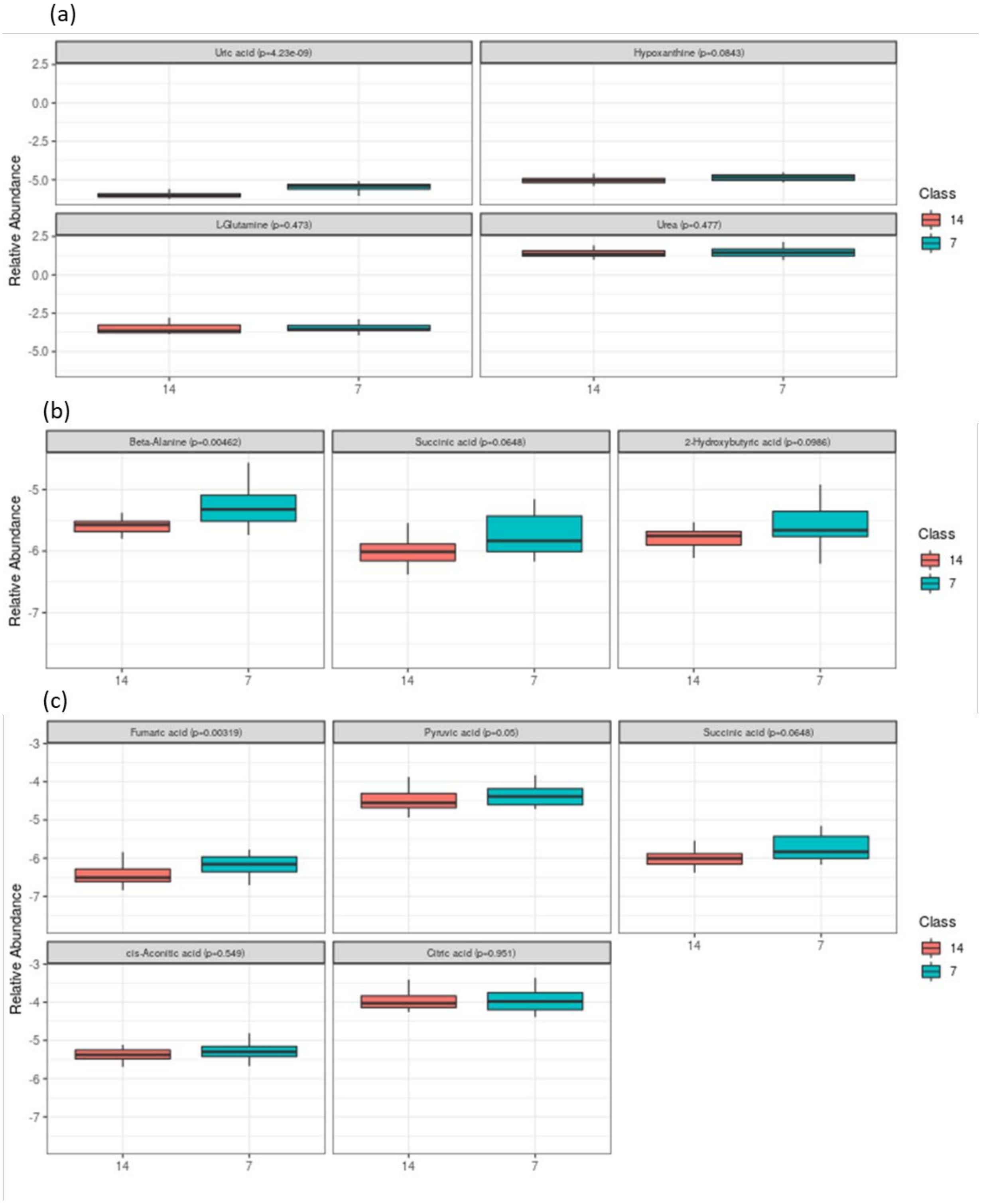

Figure S1. Influential and/or differential plasma metabolites of the top three enriched pathways comparing the water-soluble metabolites in piglets between day 7 and day 14 of life; identified using sparse partial least squares-discriminant analysis. Pathway analysis used the KEGG Pathway Database for *Homo sapiens* as a reference pathway library because no respective database was available for *Sus scrofa*. Influential and/or differential metabolites for (**a**) purine metabolism; (**b**) propanoate metabolism; and (**c**) tricarbon cycle.

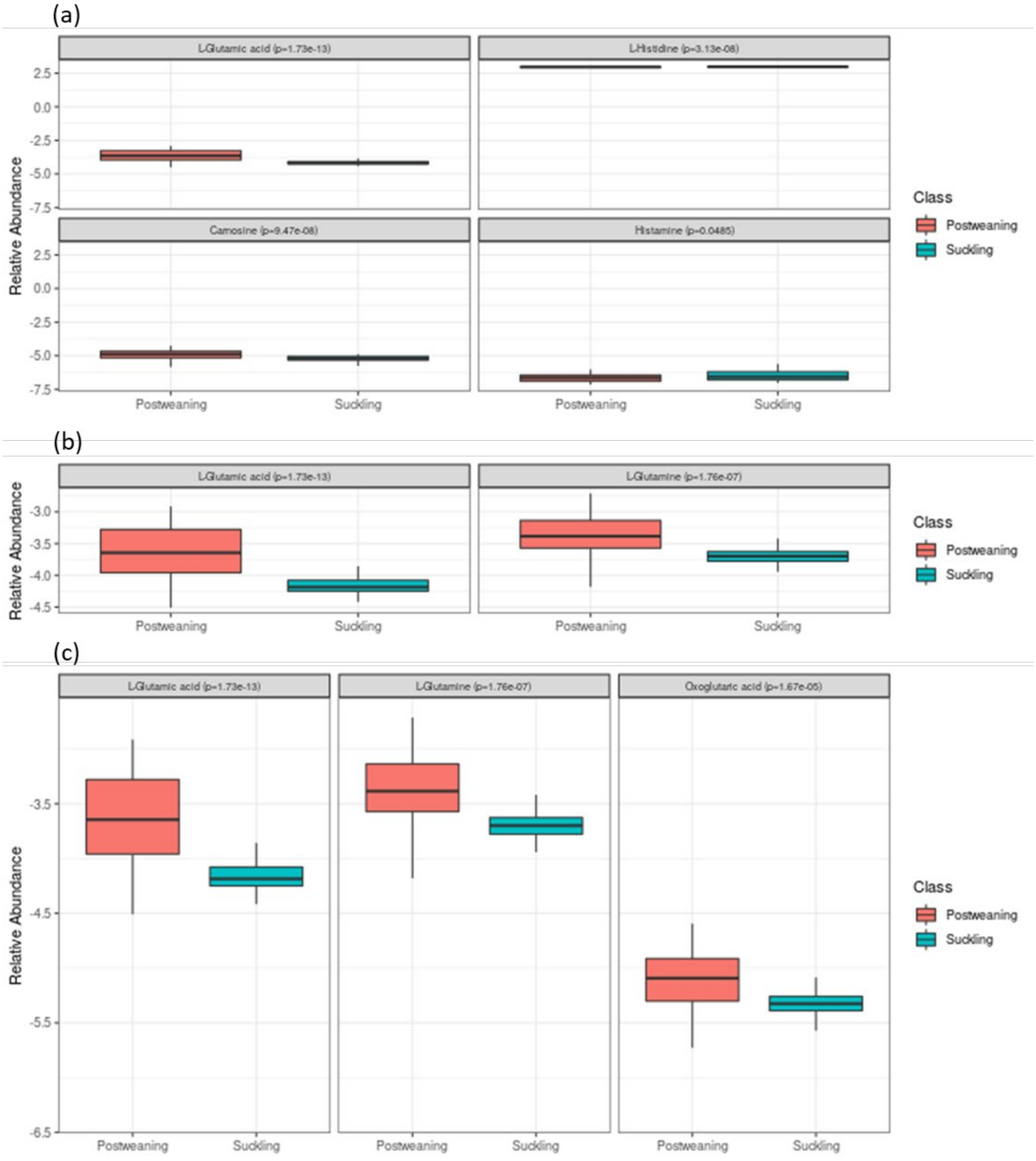

Figure S2. Influential and/or differential metabolites of the top three enriched pathways comparing the water-soluble plasma metabolites in piglets between the suckling and post-weaning period; identified using sparse partial least squares-discriminant analysis. Pathway analysis used the KEGG Pathway Database for *Homo sapiens* as a reference pathway library because no respective database was available for *Sus scrofa*. Influential and/or differential metabolites for (**a**) histidine metabolism; (**b**) nitrogen metabolism; and (**c**) d-glutamine and d-glutamate metabolism. Creep feed was fed from day 10 of life until weaning (day 28 of life).

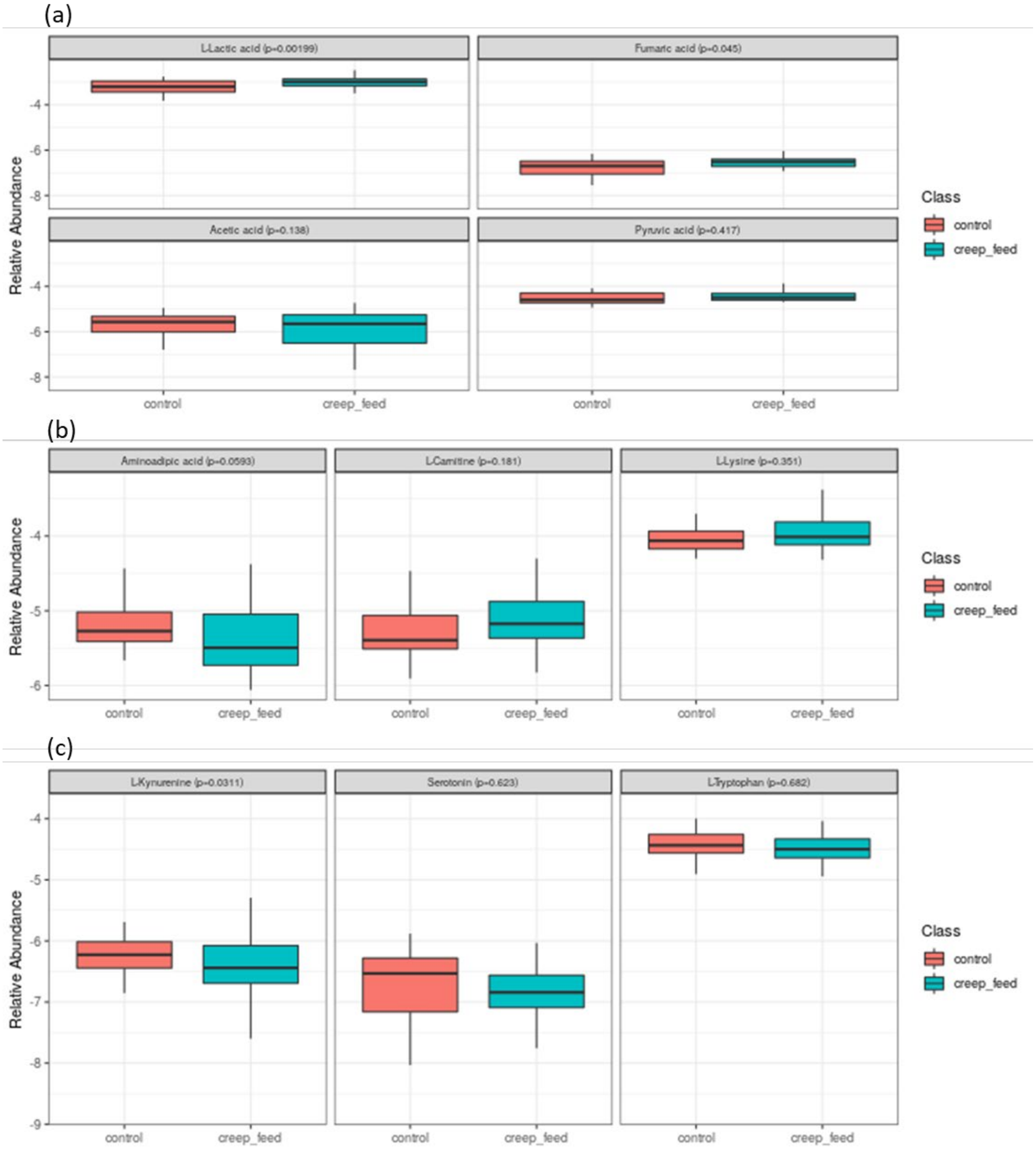

Figure S3. Influential and/or differential metabolites of the top three enriched pathways comparing the water-soluble plasm metabolites between piglets only fed sow milk and piglets receiving additional creep feed during the suckling period; identified using sparse partial least squares-discriminant analysis. Pathway analysis used the KEGG Pathway Database for *Homo sapiens* as a reference pathway library because no respective database was available for *Sus scrofa*. Influential and/or differential metabolites for (**a**) pyruvate metabolism; (**b**) lysine metabolism; and (**c**) tryptophan metabolism. Creep feed was fed from day 10 of life until weaning (day 28 of life).

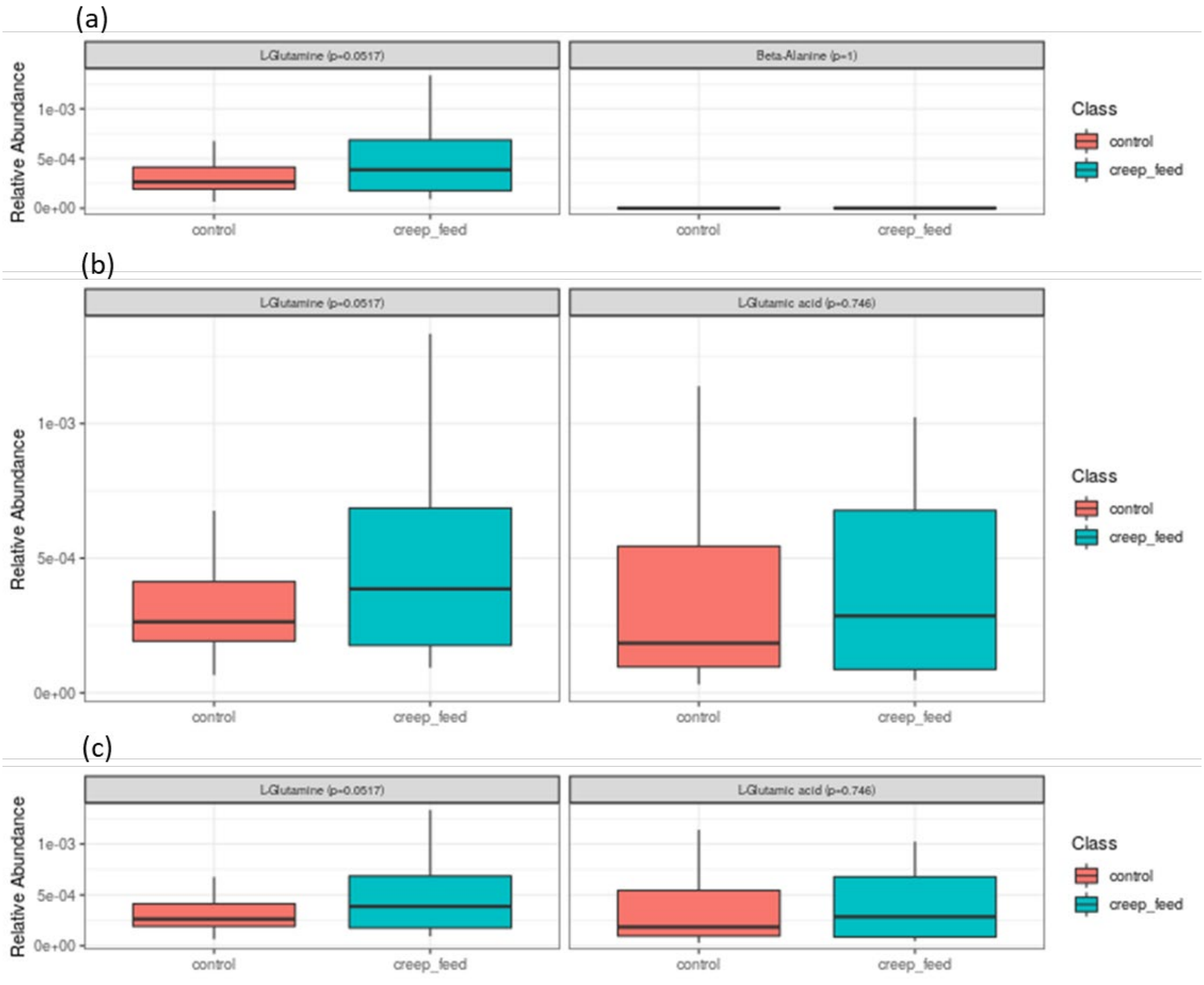

Figure S4. Influential and/or differential metabolites of the top three enriched pathways comparing the water-soluble plasma metabolites between piglets only fed sow milk and piglets receiving additional creep feed during the postweaning period; identified using sparse partial least squares-discriminant analysis. Pathway analysis used the KEGG Pathway Database for *Homo sapiens* as a reference pathway library because no respective database was available for *Sus scrofa*. Influential and/or differential metabolites for (**a**) pyrimidine metabolism; (**b**) D-glutamate and D-glutamine metabolism; and (**c**) nitrogen metabolism. Creep feed was fed from day 10 of life until weaning (day 28 of life).

Disclaimer/Publisher's Note: The statements, opinions and data contained in all publications are solely those of the individual author(s) and contributor(s) and not of MDPI and/or the editor(s). MDPI and/or the editor(s) disclaim responsibility for any injury to people or property resulting from any ideas, methods, instructions or products referred to in the content.
